# Phenotypic complexity and evolvability in evolving robots

**DOI:** 10.3389/frobt.2022.994485

**Published:** 2022-10-04

**Authors:** Nicola Milano, Stefano Nolfi

**Affiliations:** Institute of Cognitive Sciences and Technologies, CNR, Rome, Italy

**Keywords:** evolutionary robotics, complexity, evolvability, elastic soft-robots, evolving morphologies

## Abstract

The propensity of evolutionary algorithms to generate compact solutions have advantages and disadvantages. On one side, compact solutions can be cheaper, lighter, and faster than less compact ones. On the other hand, compact solutions might lack evolvability, i.e. might have a lower probability to improve as a result of genetic variations. In this work we study the relation between phenotypic complexity and evolvability in the case of soft-robots with varying morphology. We demonstrate a correlation between phenotypic complexity and evolvability. We demonstrate that the tendency to select compact solutions originates from the fact that the fittest robots often correspond to phenotypically simple robots which are robust to genetic variations but lack evolvability. Finally, we demonstrate that the efficacy of the evolutionary process can be improved by increasing the probability of genetic variations which produce a complexification of the agents’ phenotype or by using absolute mutation rates.

## 1 Introduction

One known characteristic of evolutionary algorithms consists in the tendency to discover simple solutions. One of the first demonstrations of this property has been reported in (Cliff et al., 1993) in which the authors evolved the sensory system and the neural network controller of robots selected for the ability to approach triangular objects while avoiding rectangular ones. The evolved robots relied on only two pixel photoreceptors to solve the task. Other demonstrations of this property can be derived from the experiments carried out by evolving robots constituted by rigid segments attached through actuated joints (Sims, 1994; Lipson and Pollack, 2000; Auerbach and Bongard, 2014; Auerbach et al., 2014). Indeed, in all these works the evolved solutions consist of robots formed by few segments and few actuated joints.

The tendency to generate simple solutions has advantages and disadvantages. On one hand, compact solutions can be cheaper, lighter, and/or faster than less compact ones. On the other hand, compact solutions might lack evolvability, i.e. might be harder to improve as a result of genetic variations. We hypothesize that such limited evolvability can explain why the complexity of the problems addressed by co-evolving the body and the brain of robots did not scale up significantly over the last 25 years (Cheney et al., 2016) and we propose a method for overcoming this limitation.

For this purpose, we consider the case of evolving soft robots with varying morphology. We demonstrate that the tendency to select robots with simple phenotypes reduces the evolvability of the evolving agents, i.e. the propensity to discover better solutions as a result of genetic variations, and leads to suboptimal solutions. We demonstrate that the tendency to select compact solutions originates from the fact that high performing agents often correspond to phenotypically simple robots which are more robust to genetic variations than phenotypically complex robots but lack evolvability. Finally, we demonstrate that the efficacy of the evolutionary process can be improved by increasing the probability of genetic variations which produce a complexification of the agents’ phenotype or by using absolute mutation rates.

## 2 Related research

For the sake of the objective of this paper we define evolvability as the propensity to improve as a result of genetic variations. Clearly, evolvability is influenced by the developmental process, i.e. by the way in which the genotypes of the evolving robots give rise to the corresponding phenotypic robots. For this reason, most of the research on the evolution of robots with varying morphologies focused on the design of effective developmental processes.

The first method that was used to evolve robots with varying morphology was proposed in the seminal work of Carl Sims (1994) who demonstrated the possibility to evolve neuro-controlled creatures formed by 3D rigid parts with varying shape assembled through fixed and actuated joints. In his method, the genotype encodes a directed graph, formed by nodes and connections (that can be recurrent), which encodes instructions for developing the phenotypic creature. The developmental process starts from a root node, which contains the instruction for synthesizing an initial body part, and continues with the synthesis of the body parts encoded in connected nodes. The nodes of the graph specify the properties of body parts and of associated neurons. The connections of the graph specify the relation of body parts with respect to previously created body parts. Later works adopted a similar method but relied on a smaller set of body parts and on a simpler set of developing instructions. In the case of Lipson and Pollack (2000), for example, the genotype consists of a list of tuples that encode vertices and body parts. The tuples encoding vertices represent points in the 3D space. The tuples encoding the body elements represent cylinders with varying resting length provided with fixed or actuated telescopic joints and associated neurons and neural connections. The developmental process is realized by building the corresponding body parts, one at a time, and by placing them in the locations of the associated vertices.

Other researchers proposed methods inspired more directly by the developmental process that characterizes natural multicellular organisms (Dellaert and Beer, 1996; Eggenberger, 1997; Joachimczak et al., 2016). One of the most interesting models of this class (Joachimczak et al., 2016) is based on 2D spherical cells, connected through elastic springs, that grow and differentiate from a single initial cell. The initial cell divides in 2 cells that then eventually divide in 4 cells and so on. The fate of cells is determined by a simple abstracted genetic regulatory network implemented in a feed-forward neural network. This genetic regulatory network receives as input the Cartesian coordinates of the cell and the signals received by nearby cells and determines whether the cell keeps existing or dies, the signals produced by the cell, whether the cell divides by producing two new cells, and eventually the relative positions of the new cells. The behavior of the agent is produced as a result of the periodic contraction and extension of the springs connecting the cells. In a related model proposed by Cheney et al. (2014), instead, the agents are formed by cubic cells of different types arranged within a 3D grid formed by 10 × 10 × 10 voxels (see also Corucci et al., 2018). The presence/absence of the cell and the type of the cell is determined by a genetically encoded neural network that receives as input the Cartesian coordinate of each voxel and determines as output the presence/absence of the cell and whether the corresponding voxel will be filled with a rigid cell, a soft cell, or a periodically contracting and expanding soft cell. Cells can also emit signals that diffuse over space and which regulate the contracting/expanding phase of nearby cells. Notice how these methods address the evolution of soft robots which are potentially more powerful and flexible than robots formed by rigid body parts only.

A second factor that can influence the evolvability of the agents is the timing of the developmental process. In the great majority of the works, including the works referenced above, the developmental process which gives rise to the phenotypic robots is completed before the robots start to interact with its environment and start to be evaluated. In natural organisms, instead, the developmental process extends over the entire life of the individuals. As demonstrated in recent experiments (Kriegman et al., 2018; Nadizar et al., 2022), continuing the developmental process while the robots interact with their environment can produce better solutions.

Finally, a third factor that can influence the evolvability of the agents is the usage of a selection bias which protects morphological innovation (Cheney et al., 2018). The theoretical hypothesis behind this method is that most morphological variations have a negative impact on behavioral performance since the variations of the morphological structure need to be accompanied by appropriate variations of the control policy. Temporarily reducing the selection pressure on recently morphologically changed individuals permits to adapt the control parameters to the new morphological structure. A related method has been proposed by Faíña A. et al. (2013), who retains genetic variations introducing additional body parts independently from the fitness of the corresponding individual. As claimed by the authors, “This decision was taken because adding a node can cause a fitness reduction in a given generation but act as a root for a higher growth in the next.” (Faíña et al., 2013, pp. 2411). In other words, also in this case the objective is to protect morphological variations which tend to be counter-adaptive in the short term but might be beneficial in the long term.

In this article we analyze the role of a fourth factor, the phenotypic complexity of the evolving robots. More specifically, we hypothesize that favoring the evolution of phenotypic complex robots permits to obtain individuals which are more evolvable and consequently permits to obtain better solutions. The rationale behind this hypothesis is that evolution tends to select phenotypically simple solutions which are more robust to genetic variations but which lack evolvability. This hypothesis is supported by evidences collected in previous studies carried by evolving digital circuits (Raman and Wagner, 2011; Milano and Nolfi, 2016). Moreover, the rationale of this hypothesis is in line with the idea of re-use (Anderson, 2010; Wagner, 2011; Nolfi, 2021), i.e. the fact that the larger the set of components and skill possessed by the evolving agents is, the higher the chance that new components and skills can be developed by re-using pre-existing components and skills is, and the smaller the number of variations required to develop new components and skills are. As far as we know, our work is the first attempt to analyze the role of this factor in the context of evolving robots and more specifically in the context of evolving robots with varying morphology.

## 3 Method

**FIGURE 1:**
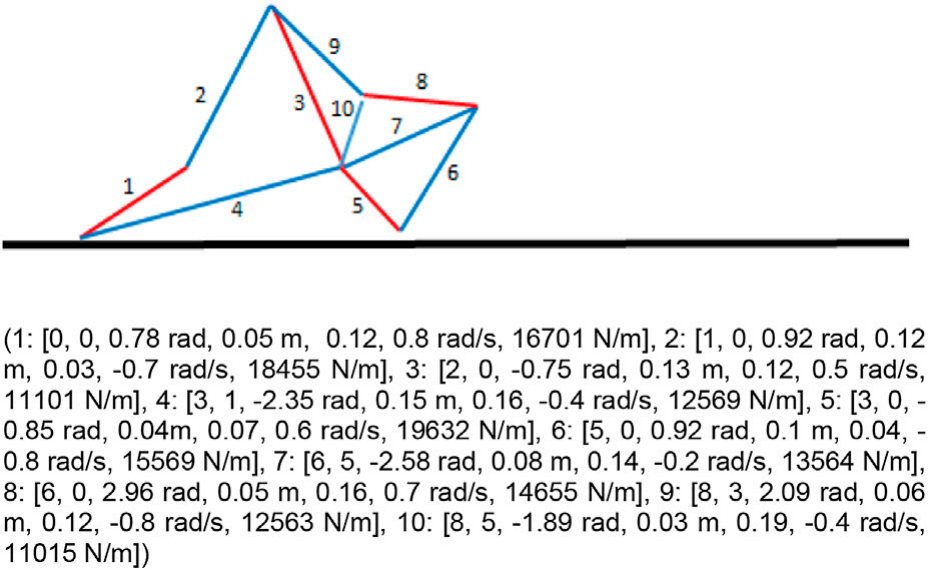
Example of a robot constituted by 10 springs. The bottom and top part of the figure shows the genotype and the phenotype of the robot. The genotype is composed of a list of tuples. Each tuple includes 7 parameters that encode the index of the starting and the ending point of spring, the angle of the spring relative to the previous element, the length, the actuation, the oscillation phase and the stiffness of the spring (see also text). The top part of the Figure shows the corresponding phenotypic robot. The black line indicates the plane. The blue and red lines indicate the spring. The two colors indicate the springs which are currently expanding and contracting, respectively. The numbers indicate the ID of the corresponding genetic tuples.

To investigate the role of phenotypic complexity in evolving robots we considered the case of 2D soft-robots made of multiple elastic springs with variable resting length which are evolved for the ability to locomote as fast as possible over a flat terrain (Figure 1). The number of elastic springs, their properties, and the way in which they are interconnected are encoded genetically. Consequently, the morphology of the evolving robots and the complexity of the robots’ body can vary across generations. The complexity of evolving robots can be estimated on the basis of the number of elastic springs forming the robots’ body.

The genotype of the robots consists of a list of tuples which encode the property of a corresponding list of elastic springs. The number of springs and their properties can vary as a result of mutations which add or remove tuples or which vary the parameters of the tuples. The dynamics of the robots and the interaction with the physical surface is simulated through the DiffTaichi physical simulator (Hu et al., 2019).

Each genetic tuple contains 7 parameters which encode the properties of a corresponding spring: 1) an integer value in the range [0, num_tuples] which encode the relative id of the tuple/spring from which the current spring originates, 2) an integer value in the range [0, num_tuples-2] which encode the relative ID of the tuple/spring in which the current spring ends, 3) a real value in the range [−π, π] rad which encodes the relative orientation with respect to the starting vertex, 4) a real value in the range [0.01,0.2] m which encodes the resting length of the spring, 5) a real value in the range [0, 0.2] m which encodes the maximum extension and contraction of the spring with respect to the resting length, 6) a real value in the range [−1.0, 1.0] rad/s which encodes the oscillation phase of the spring, and 7) a real value in the range [10,000, 20,000] N/m which encodes the stiffness of the spring.

The developmental process that gives rise to a phenotypic robot starts from an initial point located over the surface and is realized by creating the springs with the properties specified in the corresponding tuple one at a time (Figure 1). In the case of the first parameter, 0 indicates that the spring starts from the ending point of the previous tuple/spring (or from the starting vertex) while values greater than 0 indicate that the spring starts from the ending point of the corresponding preceding tuple/spring. For the second parameter, 0 indicates that the spring ends in the point located at the distance and orientation specified in the 3rd and fourth parameters of the tuple (length and angle) while values greater than 0 indicates that the spring ends in the ending point of the corresponding preceding tuple/spring. In the latter case, the 3rd and fourth parameters of the tuple are ignored and the orientation and the resting length of the spring is set on the basis of the relative position of the starting and ending points. The coordinates of the initial and ending points of the springs are perturbed with random values selected with an average of 0.0 m and a distribution of 0.02 m. These variations are introduced to emulate the variabilities characterizing physical environments.

The method used is thus similar to that introduced by Lipson and Pollack (2000). However, it is applied to evolve robots formed by elastic segments instead of rigid cylinders.

The initial population consists of N list of tuples generated randomly. The number of tuples included in each genotype is chosen randomly in the [10, 20] range, with a uniform distribution. The parameters of the tuples are generated randomly with a uniform distribution within the corresponding ranges. In the case of the first two parameters, instead, the probability to generate 0 and above 0 values are 75% and 25%, respectively. The maximum number of tuples is constrained to 32 since the DiffTaichi simulator become unstable with larger structures.

The robots are evolved by using a steady-state (*µ* + *λ*) evolutionary strategy (Rechenberg, 1973; Beyer and Schwefel, 2002) in which the best *µ* robots of the current generation are preserved in the next generation and are allowed to produce *λ* offspring each. We ran two series of experiments by using a (*1* + *4*) and a (*20* + *20*) evolutionary strategy. The former is commonly used to evolve digital circuits and graph structures (Miller et al., 2000; Miller and Thomson 2000; Miller, 2011). The latter is commonly used to evolve robots (Pagliuca et al., 2018).

During reproduction each tuple is mutated with a probability of 10%. Mutations are realized by applying one of the following genetic operators: 1) a value-change operator which replaces a randomly selected parameter of the tuple with a new value, 2) a delete operator which eliminate the tuple, or 3) an add operator which add a new tuple with randomly generated parameters after the current tuple. In the case of the mutate operator the new value of the parameter is chosen randomly within the corresponding range with a uniform distribution with the exception of the two parameters of tuples which are replaced with 0 and above 0 values with a probability of 75% and 25%, respectively. The values of the other parameters are generated randomly with a uniform distribution. We repeated the experiments by using a standard evolutionary strategy and a complexity-biased evolutionary strategy. In the former case, the probability to choose each of the three genetic operators is the same. In the latter case, instead, the probability to select the three genetic operators is [25%, 25%, 50%], respectively. Notice that this implies that in the latter case the mutations which produce a complexification of the agents’ phenotype are introduced more often than the mutations which produce a simplification of the agents’ phenotype.

The resting length of the springs varies periodically and is computed on the basis of the following equation:

y=a∙sin(t+φ);

where t is the time step, a is the maximum contraction/expansion of the spring, and φ is the phase of the spring. The latter two parameters are encoded in the genotype of the corresponding robot.

The length of the elastic spring is modified on the basis of the following equation:

$$x=x_{c}∙\left(1+y\right);$$

where x_c_ is the current length of the spring and y the sinusoidal deformation of the spring, which is encoded genetically. For a detailed description of the equations behind the physics of the simulator see (Hu et al., 2019).

The robots are evaluated for 3 episodes lasting 2000 steps. The fitness is computed by measuring the distance between the initial and final position of the center of mass of the robot averaged over the episodes. The evolutionary process is continued until the total number of steps exceeds 24 10^7^.

## 4 Results

The analysis of the results indicates that, as expected, the complexity-biased algorithm produces robots which are phenotypically more complex than the robots produced with the standard algorithm. Moreover, the results indicate that the robots evolved with the complexity-biased algorithm outperform the robots generated with the standard algorithm. This both in the experiments performed with the (20 + 20) and (1 + 4) evolutionary strategies.

**FIGURE 2:**
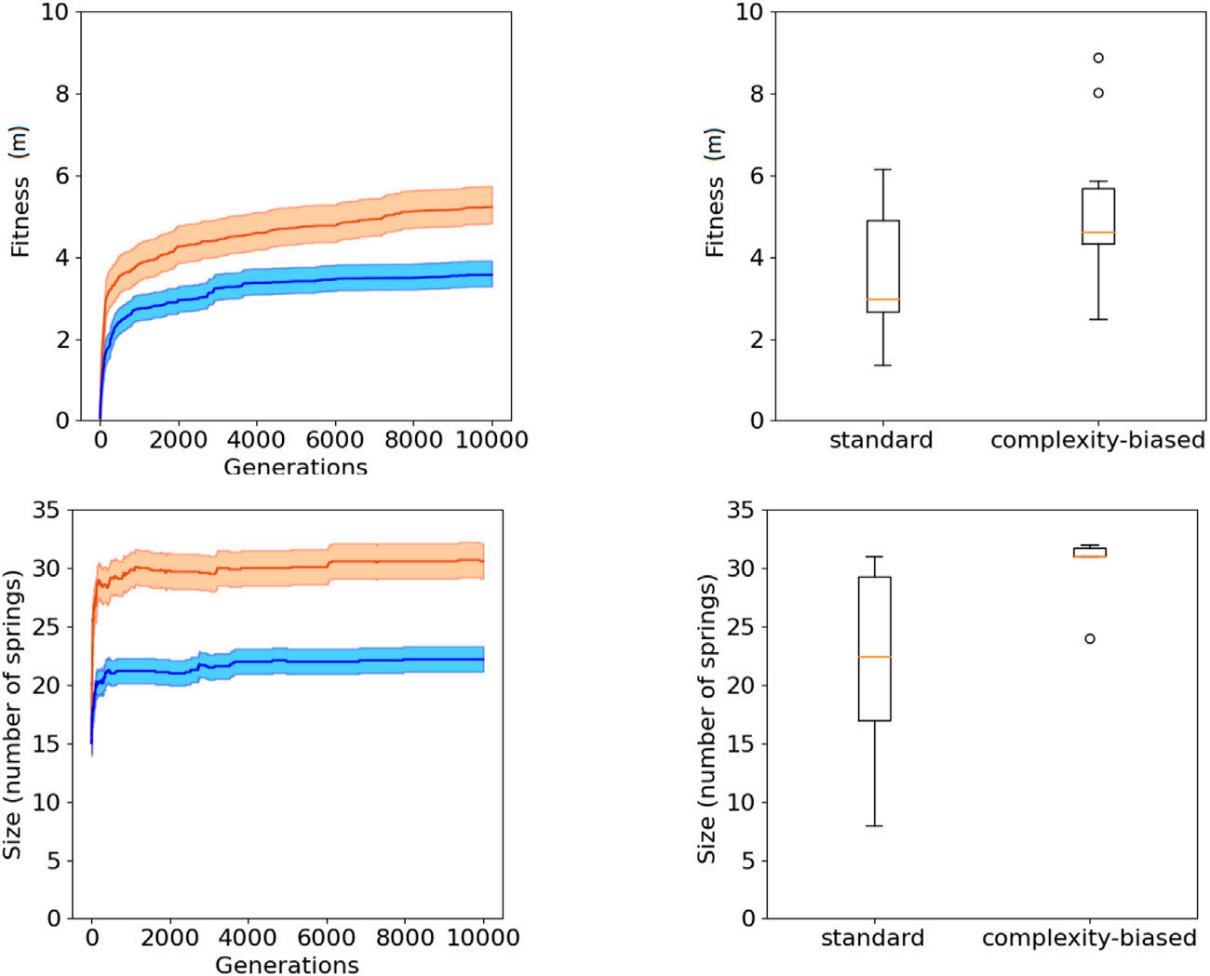
Fitness (top) and phenotypic complexity (bottom) in the case of the experiments performed with the (20 + 20) evolutionary strategy. The data shown in blue and red correspond to the experiments performed with the standard and complexity-biased algorithm, respectively. Data collected by running 10 replications of each experiment. Mean and 75% bootstrapped confidence intervals of the mean (shadow area) across 10 replications. The top-left figure shows the performance of the best individuals of each generation, averaged over 10 replications. The top-right figure shows the distribution of performance of the best 10 robots obtained in 10 corresponding replications of the experiment. The bottom-left figure shows the average phenotypic size of the best robots across generations. The bottom-right figure shows the distribution of phenotypical size of the 10 best robots obtained in the 10 corresponding replications of the experiment. The boxes represent the interquartile range of the data and horizontal lines inside the boxes mark the median values. The whiskers extend to the most extreme data points within 1.5 times the inter-quartile range from the box.

**FIGURE 3:**
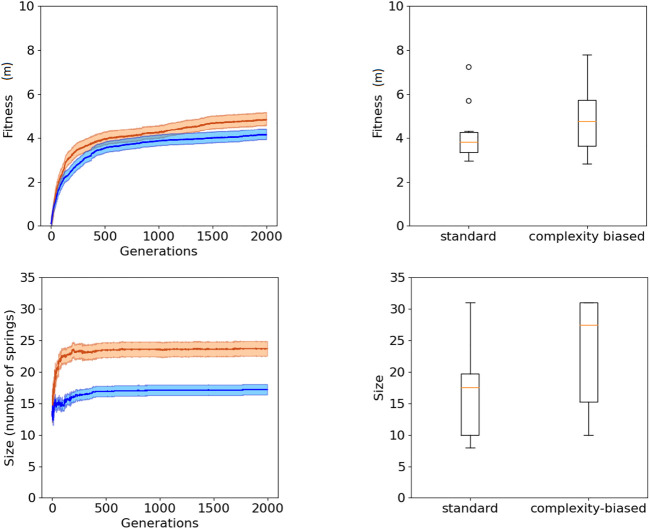
Fitness (top) and phenotypic complexity (bottom) in the case of the experiments performed with the (1 + 4) evolutionary strategy. The data shown in blue and red correspond to the experiments performed with the standard and complexity-biased algorithm, respectively. Data collected by running 10 replications of each experiment. Mean and 75% bootstrapped confidence intervals of the mean (shadow area) across 10 replications. The top-left figure shows the performance of the best individuals of each generation, averaged over 10 replications. The top-right figure shows the distribution of performance of the best 10 robots obtained in 10 corresponding replications of the experiment. The bottom-left figure shows the average phenotypic size of the best robots across generations. The bottom-right figure shows the distribution of phenotypical size of the 10 best robots obtained in the 10 corresponding replications of the experiment. The boxes represent the interquartile range of the data and horizontal lines inside the boxes mark the median values. The whiskers extend to the most extreme data points within 1.5 times the interquartile range from the box.

The fact that the robots generated with the complexity-biased algorithm are phenotypically more complex than those generated with the standard algorithm is shown by the fact that the number of springs of the best robots obtained with the former algorithm is greater than the number of spring of the best robots obtained with the latter algorithm (bottom of Figure 2 and Figure 3, Wilcoxon non parametric test *p*-value < 0.01 in both cases, sample size = 20).

The fact that the complexity-biased algorithm produces better solutions than the standard algorithm is shown by the fact that the fitness of the robots obtained with the former algorithm is greater than the fitness of the robots obtained with the latter algorithm (top of Figure 2 and Figure 3, Wilcoxon non parametric test *p*-value < 0.01 in both cases, sample size = 20).

The robots obtained with the (20 + 20) and (1 + 4) evolutionary strategies do not differ significantly in term of performance and phenotypic complexity (Wilcoxon non parametric test *p*-value > 0.05, sample size = 20).

**FIGURE 4:**
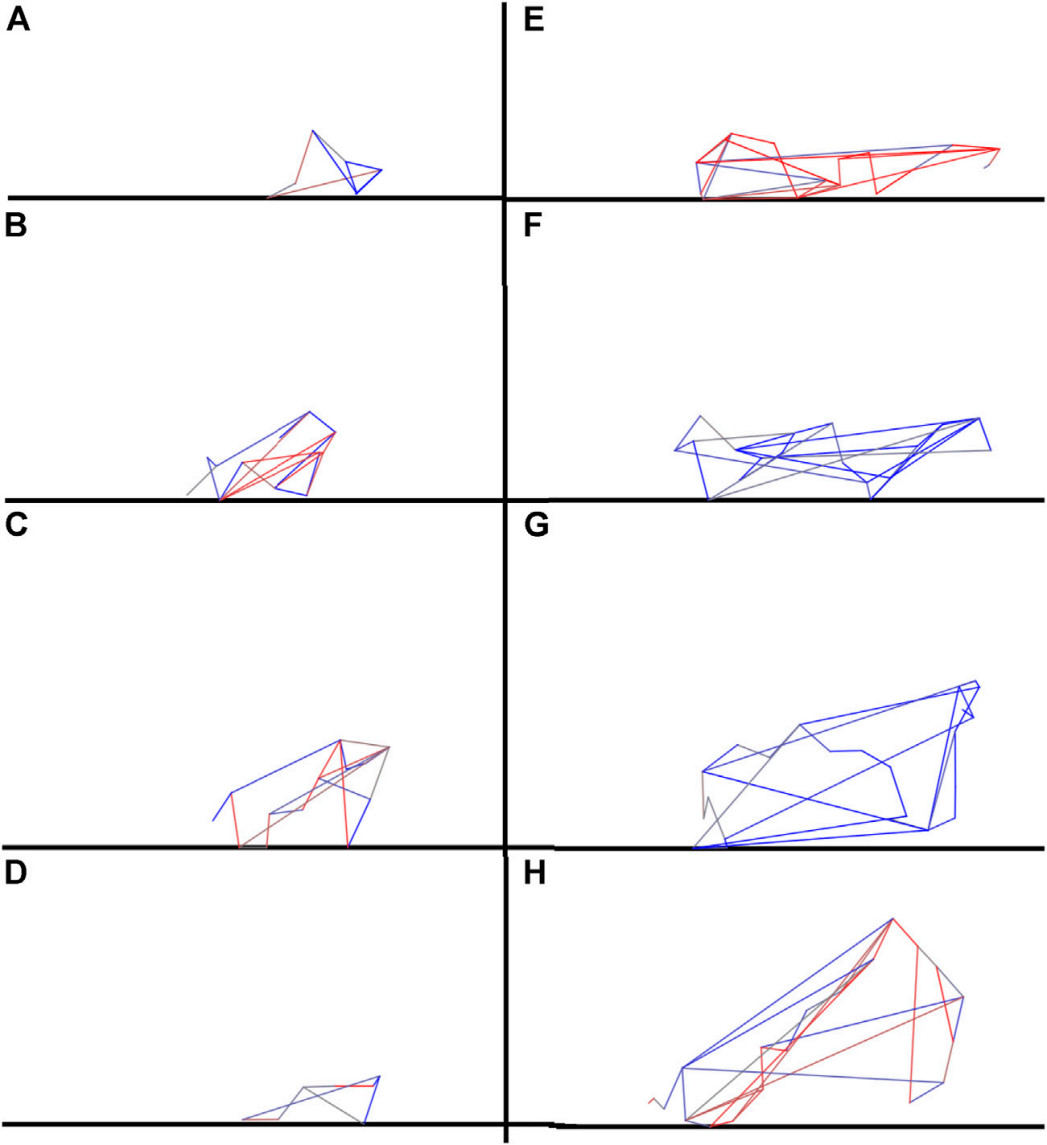
Examples of robots evolved with the standard and complexity-biased algorithm, left **(A–D)** and right respectively **(E–H)**. The agents shown were selected among the best performing agents. The link to the videos of the corresponding behaviors are included in the Appendix A. The colored lines indicate the springs forming the robot’s body. The colors indicate the contraction/extension phase of the springs.

The qualitative difference among the robots obtained with the standard and complexity-biased algorithms can also be appreciated by visually comparing the morphologies (Figure 4) and the behaviors (see the appendix) exhibited by the robots evolved with the standard and complexity-biased algorithm.

The advantage of the complexity-biased algorithm can be explained by assuming that the variations increasing phenotypic complexity provide an adaptive advantage in the long term but not in the short term. In other words, it can be explained by assuming that the variations which increase the complexity of the phenotype improve the evolvability of the agents.

**FIGURE 5:**
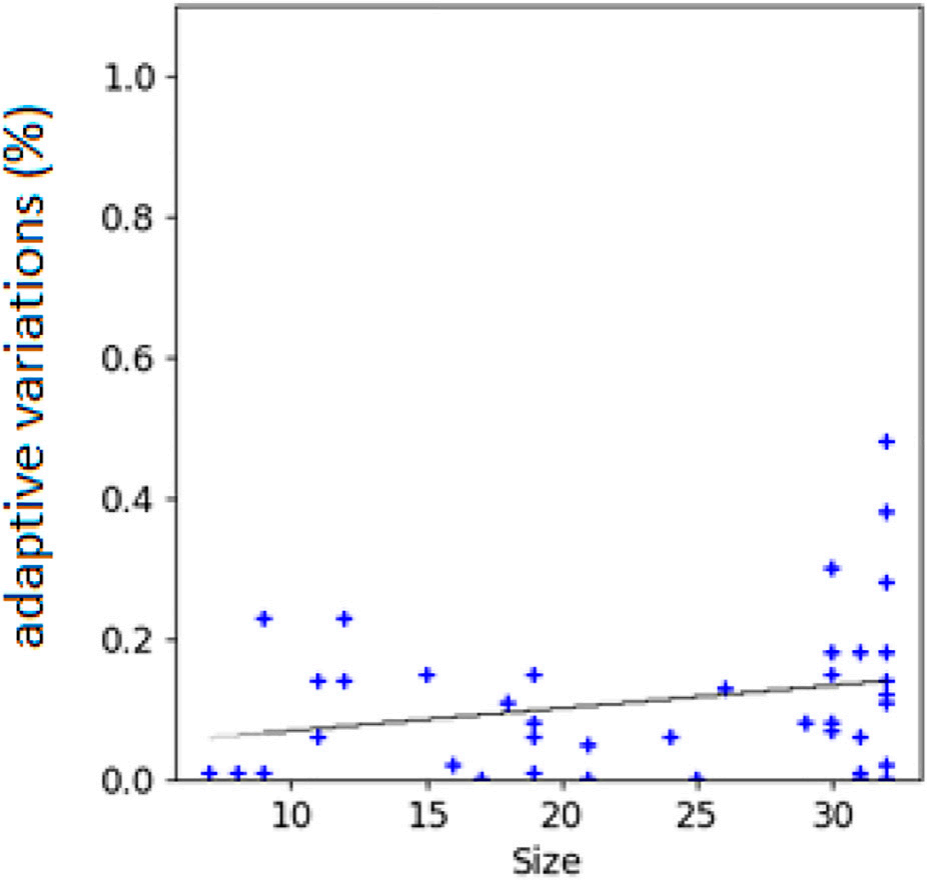
Correlations between the size of the robots’ phenotype and the percentage of adaptive variations. Data computed by subjecting the agents of intermediate generations to single genetic variations for 1000 times. Intermediate generations refer to generation totg/2, where totg correspond to the total number of generations. The black line indicates the linear interpolation of the data. Data obtained by analyzing 40 robots evolved with the (20+20) and (1+4) evolutionary strategy and evolved with the standard and complexity-biased algorithm (i.e. the robots produced in the four experimental conditions, 10 replications for each condition).

**FIGURE 6:**
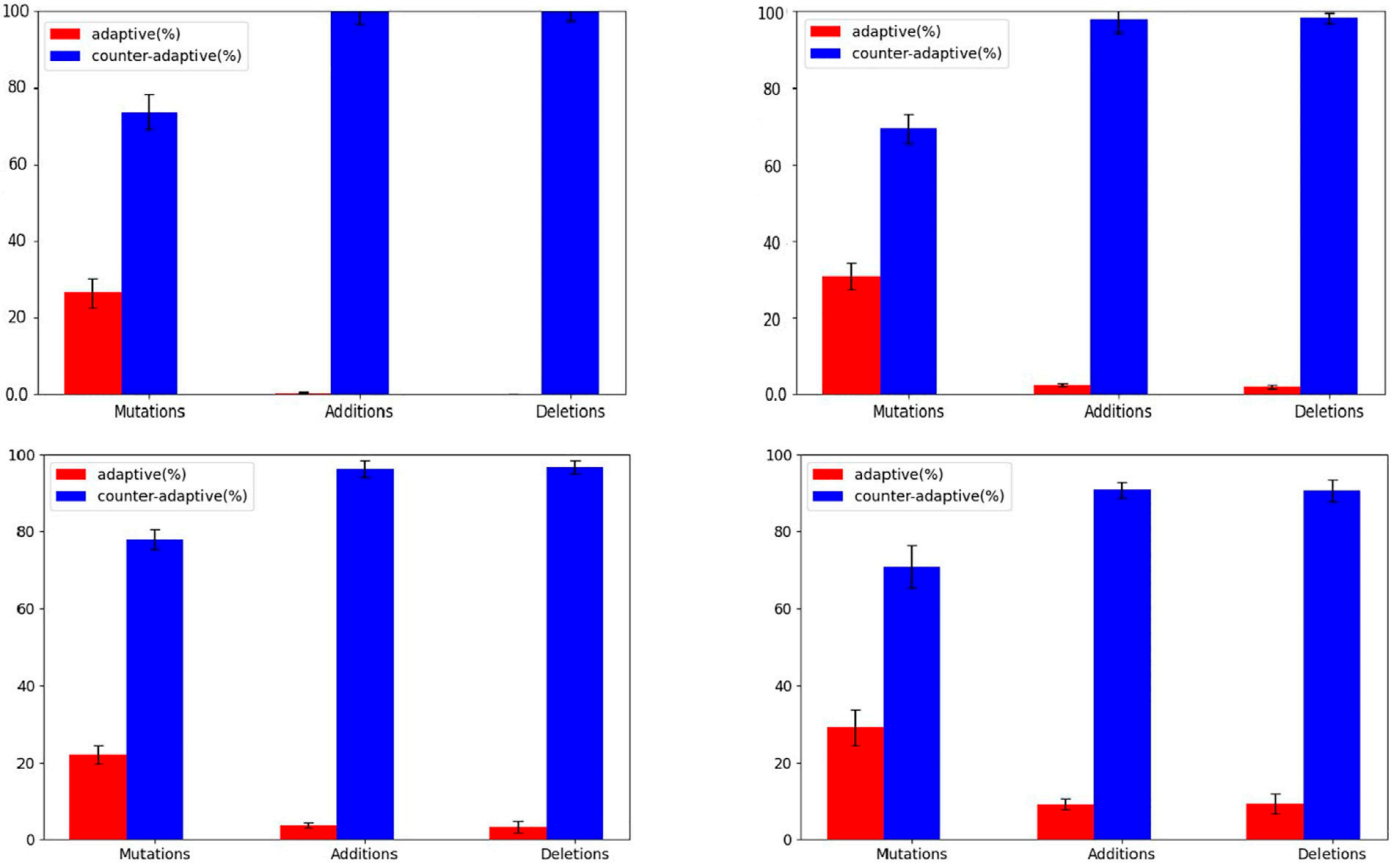
Fraction of adaptive and counter-adaptive value-change, addition, and deletion mutations. The top and bottom figures display the results obtained with the (1+4) and (20+20) algorithms, respectively. The left and right figures display the results obtained with the standard and complexity-biased algorithms, respectively. Data calculated on the best evolving individual of an intermediate generation, i.e. of the generation which corresponds to the half of the total number of generations. Data averaged over 20 individuals extracted from 20 corresponding replications of the experiment. The fraction of adaptive and maladaptive mutation is calculated by verifying whether the fitness of the individual increases or decreases after the introduction of a single value-change or addition or deletion mutation. For this analysis the agents are evaluated for 10 episodes. Each individual is subjected to 1000 genetic variations of each kind. The fraction of neutral genetic variations is negligible.

The hypothesis is confirmed by the analysis of the correlation between the size of the evolving agents and the probability that mutations are adaptive or counter-adaptive. Indeed, the percentage of mutations that result adaptive correlates positively with the size of the phenotype of the individuals (Figure 5, Pearson correlation coefficient: 0.35). Moreover, the hypothesis is confirmed by the analysis of the different types of genetic variations displayed in Figure 6. Indeed, the fraction of variations which result adaptive is greater in the agents evolved with the complexity-biased algorithm which produce phenotypically more complex individuals (Wilcoxon non parametric test *p*-value < 0.01 in both cases, sample size = 40). Notice in particular, that the fraction of additions and deletions which result adaptive in the case of the experiment performed with the (20 + 20) evolutionary strategy is small but significant in the case of the complexity-biased algorithm while is negligible in the case of the standard algorithm. We will discuss the difference between the (20 + 20) and (1 + 4) evolutionary strategies below. A positive correlation between evolvability and size was already found in digital circuits by Raman and Wagner (2011) and by us (Milano and Nolfi, 2016). As far as we know, this is the first time that the correlation is observed in evolving robots.

Notice that the property that influences the evolvability of the agents in these experiments differs from the property discussed in previous studies (Wagner and Altenberg, 1996). In this work the authors hypothesize a relation between evolvability and modularity, defined as a genotype-to-phenotype mapping ensuring a reduced pleiotropy between genes responsible for different functions. In this study, instead, we found a relation between evolvability and phenotypic complexity, defined as the number of elementary parts forming the agents’ body. Both properties can play a role and future studies might investigate the relative weight of the two properties. We do not analyze this issue here since our experimental setting is probably inadequate to study the role of modularity.

At this point we might wonder why the phenotypic complexity of the evolving agents do not keep increasing across generations despite phenotypical complex individuals achieve higher performance and are more evolvable (i.e. have a higher propensity to generate better solutions as a result of mutations) than phenotypically simple individuals. In the context of our experimental setting, we might wonder why additions, which produce phenotypically complex individuals, are not retained more often than deletions, which produce phenotypically simple individuals.

A possible explanation is that the advantages provided by additions with respect to deletions is too weak to ensure a significantly different retention rate of additions with respect to deletions. However, a weak advantage should produce at least a small difference in the survival rate of the individuals. The fact that the difference is small should have an impact on the rate with which individuals complexify across generations but should lead to a progressive complexification which does not manifest in our experiments.

A second possible explanation is that the advantages granted by additions are compensated by a disadvantage caused by a second factor. Such a second factor can be the robustness to genetic variations, i.e. the propensity to generate offspring who maintain the performance level of the parent (who do not perform poorer than the parent). Such second factor is caused by the fact that the offspring of phenotypically simple individuals undergo a smaller number of variations, on the average, than the offspring of phenotypically complex individuals. Mutations are counter-adaptive in most of the cases (see Figure 6). Consequently, the smaller the number of mutations received, the higher the chances that the individual preserves a performance level similar to the parent is. This hypothesis was already demonstrated in the context of the evolution of digital circuits (Milano and Nolfi, 2016; Milano, Pagliuca and Nolfi, 2019; Milano and Nolfi, 2021). In this article we demonstrate for the first time that this factor also constraints the evolution of morphologies in evolving robots.

Interestingly, this explanation implies that the chance to retain additions and deletions should depend on whether each tuple has a certain probability to be mutated or whether the number of mutations is fixed. Indeed, in the former case we should expect a correlation between phenotypic complexity and robustness to genetic variations. In the latter case, instead, the robustness to genetic variations should be independent from the phenotypic complexity of the individuals.

To verify this prediction, we carried out additional experiments in which we mutated a single tuple, independently from the length of the genotype, and in which we used different mutation rates (2%, 5% and 10%). For each condition we ran 10 replications of the experiments. The obtained results confirm that mutating a single tuple produces phenotypically larger individuals and leads to better performance (Table 1), in the case of the (20 + 20) evolutionary strategy. In the experiments in which each tuple is mutated with a probability of 2%, 5%, or 10%, the mutation rate has an effect on performance and does not have an effect on the size of the evolving individuals. As expected, the best performance is obtained with an intermediate mutation rate which maximizes the exploration/exploitation ratio.

**TABLE 1:** Performance obtained by introducing a single mutation and by varying the mutation rate. The values included in round brackets show the average and the standard deviation of performance. The values shown in square brackets indicate the average and the standard deviation of size. Results obtained with the standard (20+20) and (1+4) algorithms. The experiments performed by using a mutation rate of 10% are the same experiments reported above.

Algorithm	Single Mutation	Mutation rate 2%	Mutation rate 5%	Mutation rate 10%
(20+20) ES	(4.7 ± 0.6) [25.3± 7.5]	(3.6 ± 0.7) [17 ± 8.6]	(4.2 ± 0.7) [16.9 ± 9.3]	(3.5 ± 0.9) [16.2 ± 8.8]
(1+4) ES	(3.2 ± 1.9) [23.2 ± 8.3]	(3.1 ± 1.7) [24.4 ± 7.2]	(3.7 ± 1.9) [24.1 ± 7.5]	(3.2 ± 1.6) [23.1 ± 8.3]

Before concluding this section, we should discuss the difference between the (20 + 20) and the (1 + 4) evolutionary strategies. The results reported in Figure 2 and Figure 3 and in Table 1 show that the (1 + 4) evolutionary strategy, which generates the population from a single parent, produces individuals which are phenotypically more complex than the individuals generated with the (20 + 20) evolutionary strategy. This can be explained by considering that in the case of the latter algorithm, the offspring of the agents which are phenotypically simpler have more chance to survive than the offspring of the agents which are phenotypically more complex. In other words, the competition among the lineages generated by multiple parents favors phenotypically simpler agents which are more robust to mutations. The only exception is the case of the experiment performed by introducing a single mutation. In this case in fact, the genetic robustness of the individuals does not depend on the complexity of the phenotype.

The (1 + 4) evolutionary strategy differs in that respect since the presence of a single parent eliminates the competition among multiple lineages. This explains why in the case of the (1 + 4) evolutionary strategy, mutating a single tuple or mutating each tuple with a certain probability does not have an impact on the size of the evolving individuals. As mentioned above, the (1 + 4) evolutionary strategy produces individuals which are genotypically more complex than the individuals generated with the (20 + 20) evolutionary strategy. On the other hand, the (20 + 20) evolutionary strategy produces better results in settings in which the phenotypic complexity is not constrained by the necessity to be robust to genetic variations, like in the single mutation setting.

## 5 Conclusion

In this work we studied the relation between phenotypic complexity and evolvability in the case of soft-robots with varying morphology. Moreover, we discussed the mechanisms which can favor the evolution of evolvable robots and consequently which can lead to better solutions.

We started our analysis by demonstrating that the phenotypic complexity of the robots, estimated by counting the number of body parts forming the robots’ body, correlates positively with the evolvability of the robots, defined as the propensity of the evolving robots to improve as a result of genetic variations. Previous works hypothesized a correlation between evolvability and modularity, defined as reduced pleiotropy among genes serving different functions (Wagner and Altenberg, 1996). As far as we know this is the first time that a link between evolvability and phenotypic complexity has been pointed out in the context of evolving robots.

We then demonstrated that evolution does not necessarily select phenotypical-complex robots characterized by high evolvability. On the contrary, evolution remains stuck on robots with relatively simple morphologies which are suboptimal and which have a low evolvability. This is caused by the relationship between phenotypic complexity and robustness to genetic variations. Indeed, the longer the number of genes playing a function is, the higher the chance that mutations modify the characteristics of the offspring with respect to the parent is, and the higher the chance that the offspring is less fit than the parent is.

Notice that the negative correlation between phenotypic complexity and robustness to genetic variations affect only the settings in which the number of genes can vary across generations. This is typically the case of the experiments in which the morphology of the evolving robots and/or the architecture of the robots’ neural controllers vary evolutionarily. In the experiments in which the morphology of the robot and the architecture of the robots’ neural controller is fixed and in which the only parameters that are subjected to variations are the connection weights, the phenotypic complexity of the robots’ phenotype remains constant. This is probably the reason why the evolution of robots with fixed architecture has been scaled to significant more complex problems over the years (see for example Salimans 2017) while the study of the evolution of robots with varying morphology remained stuck on relatively simple agents and problems (Cheney et al., 2016).

Finally, we demonstrated that the problem described above can be ameliorated by: 1) introducing genetic variations leading to a complexification of the agents’ phenotype more often than variations leading to a simplification, 2) introducing a fixed number of variations, and 3) using evolutionary algorithms which generate the population on the basis of a single parent. The first strategy operates by introducing a bias that acts directly on the complexification of the robots’ phenotype. The second strategy eliminates the correlation between the length of the genotype (and of the phenotype) and the robustness to genetic variations. The third strategy eliminates the competition between alternative lineages which favors the selection of phenotypically simple individuals.

Algorithms fostering the selection of novel solutions or the generation of population including diversified solutions (Lehman and Stanley, 2011; Mouret and Clune, 2015; Nordmoen et al., 2021) can also contrast the tendency to remain stuck on phenotypic simple solutions. Consequently, the efficacy of these algorithms can be interpreted at least in part to their ability to overcome the problem discussed above.

The relative efficacy of these strategies and the possibility to combine the advantages of multiple strategies should be analyzed in future studies.

The relation between robustness to genetic variations and phenotypic complexity can depends on the genotype-to-phenotype relationship. More specifically, it could be weaker or absent in experimental settings in which the relation between the genotype and the phenotype is more indirect. Future research should verify this aspect. The comprehension of this aspect could also be used to design genotype-to-phenotype mappings which are not biased toward phenotypical simple and low evolvable solutions.

## Data Availability

The datasets presented in this study can be found in online repositories. The names of the repository/repositories and accession number(s) can be found below: https://github.com/milnico/phenotypic_complexity.

## Appendix A:

Standard evolved morphologies examples:

https://youtu.be/Jl8QScKBZSs
https://youtu.be/Zh8jtc70WOA
https://youtu.be/9xYrA84yKPY
https://youtu.be/GgVO_wpSdpo

Complexity biased evolved morphologies examples:

https://youtu.be/48zt0gZ4qnk
https://youtu.be/qxO6sutm2YE
https://youtu.be/EnZ0XFelNnI
https://youtu.be/PXMaUU61V2A

## References

[B1] Anderson M. L. (2010). Neural reuse: A fundamental organizational principle of the brain. Behav. Brain Sci. 33 (4), 245–266. 10.1017/s0140525x10000853 20964882

[B2] Auerbach J. E. Aydin D. Maesani A. Kornatowski P. M. Cieslewski T. Heitz G. (2014). “RoboGen: Robot generation through artificial evolution,” in Proceedings of the fourteenth international conference on the synthesis and simulation of living systems (ALIFE 14). Editors Sayama H. Reiffel J. Risi S. Doursat R. Lipson H. (New York: The MIT Press).

[B3] Auerbach J. E. Bongard J. C. (2014). Environmental influence on the evolution of morphological complexity in machines. PLoS Comput. Biol. 10 (1), e1003399. 10.1371/journal.pcbi.1003399 24391483 PMC3879106

[B4] Beyer H. G. Schwefel H. P. (2002). Evolution strategies—A comprehensive introduction. Nat. Comput. 1 (1), 3–52. 10.1023/a:1015059928466

[B5] Cheney N. Bongard J. SunSpiral V. Lipson H. (2018). Scalable co-optimization of morphology and control in embodied machines. J. R. Soc. Interface 15 (143), 20170937. 10.1098/rsif.2017.0937 29899155 PMC6030623

[B6] Cheney N. Bongard J. Sunspiral V. Lipson H. (2016). “On the difficulty of co-optimizing morphology and control in evolved virtual creatures,” in Artificial Life Conference Proceedings (Cambridge, MA: MIT Press), 226–233.

[B7] Cheney N. Clune J. Lipson H. (2014). “Evolved electrophysiological soft robots,” in Proceedings of the fourteenth international conference on the synthesis and simulation of living systems (ALIFE 14). Editors Sayama H. Reiffel J. Risi S. Doursat R. Lipson H. (New York: The MIT Press).

[B8] Cliff D. Harvey I. Husband P. (1993). Explorations in evolutionary robotics. Adapt. Behav. 2, 73–110. 10.1177/105971239300200104

[B9] Corucci F. Cheney N. Giorgio-Serchi F. Bongard J. Laschi C. (2018). Evolving soft locomotion in aquatic and terrestrial environments: Effects of material properties and environmental transitions. Soft Robot. 5 (4), 475–495. 10.1089/soro.2017.0055 29985740

[B10] Dellaert F. Beer R. D. (1996). “A developmental model for the evolution of complete autonomous agents,” in From animals to animats 4: Proceedings of the 4th International Conference on Simulation of Adaptive Behavior (SAB 1996), (Cambridge, MA: MIT Press), 393–401.Eggenberger.

[B11] Eggenberger H. (1997). “Evolving morphologies of simulated 3D organisms based on differential gene expression,” in Proceedings of the 4th European Conference on Artificial Life (ECAL 1997) (Cambridge, MA: MIT Press), 205–213.

[B12] Faíña A. Bellas F. Lopez-Pena F. Duro R. J. (2013). EDHMoR: Evolutionary designer of heterogeneous modular robots. Eng. Appl. Artif. Intell. 26 (10), 2408–2423. 10.1016/j.engappai.2013.09.009

[B13] Hu Y. Anderson L. Li T. M. Sun Q. Carr N. Ragan-Kelley J. (2019). Difftaichi: Differentiable programming for physical simulation. Available at: https://arxiv.org/abs/1910.00935 .

[B14] Joachimczak M. Suzuki R. Arita T. (2016). Artificial metamorphosis: Evolutionary design of transforming, soft-bodied robots. Artif. life 22 (3), 271–298. 10.1162/artl_a_00207 27139940

[B15] Kriegman S. Cheney N. Bongard J. (2018). How morphological development can guide evolution. Sci. Rep. 8 (1), 13934–14010. 10.1038/s41598-018-31868-7 30224743 PMC6141532

[B16] Lehman J. Stanley K. O. (2011). “Evolving a diversity of virtual creatures through novelty search and local competition,” in Proceedings of the 13th annual conference on Genetic and evolutionary computation, Dublin, Ireland, 211–218.

[B17] Lipson H. Pollack J. B. (2000). Automatic design and manufacture of robotic lifeforms. Nature 406 (6799), 974–978. 10.1038/35023115 10984047

[B18] Milano N. Nolfi S. (2021). Enhancing Cartesian genetic programming through preferential selection of larger solutions. Evol. Intell. 14 (4), 1539–1546. 10.1007/s12065-020-00421-9

[B19] Milano N. Nolfi S. (2016). Robustness to faults promotes evolvability: Insights from evolving digital circuits. PloS one 11 (7), e0158627. 10.1371/journal.pone.0158627 27409589 PMC4943595

[B20] Milano N. Pagliuca P. Nolfi S. (2019). Robustness, evolvability and phenotypic complexity: Insights from evolving digital circuits. Evol. Intell. 12 (1), 83–95. 10.1007/s12065-018-00197-z

[B21] Miller J. F. (2011). Cartesian genetic programming. Belin: Springer-Verlag.

[B22] Miller J. F. Job D. Vassiley V. K . (2000). Principles in the evolutionary design of digital circuits. Genet. Program. Evolvable Mach. 1 (1), 7–35. 10.1023/a:1010016313373

[B23] Miller J. F. Thomson P. (2000). “Cartesian genetic programming,” in Lecture notes in computer science 1802 genetic programming. Editors Poli R. Banzhaf W. Langdon W. B. Miller J. Nordin P. Fogarty T. C. (Heidelberg: Springer).

[B24] Mouret J. B. Clune J. (2015). Illuminating search spaces by mapping elites. Available at: https://arxiv.org/abs/1504.04909 .

[B25] Nadizar G. Medvet E. Karine M. (2022). “On the schedule for morphological development of evolved modular soft robots,” in European Conference on Genetic Programming (Part of EvoStar), Madrid, Spain (Cham: Springer).

[B26] Nolfi S. (2021). Behavioral and cognitive Robotics: An adaptive perspective. Roma, Italy: Institute of Cognitive Sciences and Technologies, National Research Council CNR-ISTC. 10.1007/s10339-011-0402-3 21468745

[B27] Nordmoen J. Veenstra F. Ellefsen K. O. Glette K. (2021). MAP-elites enables powerful stepping stones and diversity for modular robotics. Front. Robot. AI 8, 639173. 10.3389/frobt.2021.639173 33996926 PMC8115726

[B28] Pagliuca P. Milano N. Nolfi S. (2018). Maximizing adaptive power in neuroevolution. PloS one 13 (7), e0198788. 10.1371/journal.pone.0198788 30020942 PMC6051599

[B29] Raman K. Wagner A. (2011). The evolvability of programmable hardware. J. R. Soc. Interface 8 (55), 269–281. 10.1098/rsif.2010.0212 20534598 PMC3033018

[B30] Rechenberg I. (1973). Evolutionstrategie—optimierung technischer Systeme nach Prinzipien der biologischen Evolution. Stuggart: Frommann-Holzboog.

[B34] Salimans T. Ho J. Chen X. Sidor S. Sutskever I. (2017). Evolution strategies as a scalable alternative to reinforcement learning. arXiv [Preprint]. Available at: https://arxiv.org/abs/1703.03864 .

[B31] Sims K. (1994). Evolving 3d morphology and behavior by competition. Artif. Life 1 (4), 353–372. 10.1162/artl.1994.1.4.353

[B32] Wagner A. (2011). The origins of evolutionary innovations: A theory of transformative change in living systems. Oxford: OUP.

[B33] Wagner G. P. Altenberg L. (1996). Perspective: Complex adaptations and the evolution of evolvability. Evolution 50 (3), 967–976. 10.2307/2410639 28565291

